# Efficacy and safety of cinobufacini injection combined with vinorelbine and cisplatin regimen chemotherapy for stage III/IV non-small cell lung cancer: A protocol for systematic review and meta-analysis of randomized controlled trials

**DOI:** 10.1097/MD.0000000000021539

**Published:** 2020-07-31

**Authors:** Qian Li, Ren-Long Liang, Qian-Ru Yu, De-Qing Tian, Li-Na Zhao, Wen-Wen Wang, Hua Xiao, Xiao-Jia Yong, Xiao-Dong Peng

**Affiliations:** a School of Health Preservation and Rehabilitation; b Basic Medical School, Chengdu University of Traditional Chinese Medicine; c Department of Oncology, The Second People's Hospital of Chengdu, Sichuan, China.

**Keywords:** cinobufacini injection, cisplatin, meta-analysis, non-small cell lung cancer, randomized controlled trial, vinorelbine

## Abstract

**Background:**

The main component of cinobufacini injection is dry toad skin, which is used as adjuvant therapy for stage III/IV non-small cell lung cancer patients in long-term combination with vinorelbine and cisplatin. However, the efficacy and safety of this combination therapy remain unclear.

**Methods:**

A systematic review and meta-analysis will be conducted following the preferred reported items for systematic review and meta-analysis guidelines. Two independent reviewers (LRL and ZLN) will carry out a comprehensive search of the PubMed, Web of Science, Cochrane Library, EMBASE, the Chinese Science and Technology Periodical Database, China National Knowledge Infrastructure, Wanfang Databases, China Biology Medicine. The last search date will be July 30, 2020. Reference list of all selected articles will independently screened to identify additional studies left out in the initial search. The Cochrane Risk of Bias Tool will be used to evaluate the risk of bias of the randomized controlled trials. Outcome index: The main efficacy indicators were based on the objective efficacy evaluation criteria of the World Health Organization antineoplastic drugs or the objective efficacy evaluation criteria of solid tumors established by RECIST. Secondary criteria Karnofsky performance scale (KPS) score, pain efficacy criteria, side effects of chemotherapy such as myelosuppression and gastrointestinal symptoms. Assessment of risk of bias and data synthesis will be conducted using Review Manager V5.3 software.

**Results:**

This study will systematically evaluate the efficacy and safety of cinobufacini combined with vinorelbine and cisplatin in the treatment of stage III/IV non-small cell lung cancer. The results of this systematic review will be published in peer-reviewed scientific journals.

**Ethics:**

The ethical approval is not required since systematic review is based on published studies.

**INPLASY registration number:**

INPLASY202060091.

## Abstract 2

Supplemental Digital Content is available in the text

## 1 Introduction

Lung cancer is the leading cause of cancer death among men and the second leading cause of cancer death among women worldwide.^[1]^ It is one of the malignant tumors with the highest morbidity and mortality in the world, which is a serious threat to human health.^[2,3]^ Non-small cell lung cancer (NSCLC) accounts for about 85% of all lung cancer cases. Most of the patients are in the late stage at the first visit, miss the opportunity for operation, and are easy to relapse after operation.^[4,5]^

Surgery, radiotherapy, and chemotherapy are considered to be the 3 pillars in the treatment of NSCLC. If the opportunity for surgery is lost, multidisciplinary treatment based on chemotherapy will be adopted.^[6,7]^ Chemotherapy can prolong the survival time of patients with advanced NSCLC, improve symptoms, and have a good titer ratio.^[8,9]^ Vinorelbine combined with cisplatin is a first-line regimen for patients with unresectable advanced NSCLC, but it may have side effects such as myelosuppression and gastrointestinal disorders.^[10–12]^

The treatment of traditional Chinese medicine is one of the ideal means for the treatment of advanced lung cancer in the near future, and it is effective in relieving symptoms, reducing toxicity, and increasing efficiency. The treatment of traditional Chinese medicine is mainly characterized by higher stable rate of focus, longer survival time, and better quality of life.^[13,14]^ In recent years, more and more traditional Chinese medicine injections are used.^[15,16]^ When it comes to the treatment of lung cancer, it changes the traditional administration mode of traditional Chinese medicine, which has the advantages of fast absorption, rapid action, and convenient clinical application. There are 136 kinds of traditional Chinese medicine injections registered by the National Medical Products Administration (http://www.nmpa.gov.cn/WS04/CL2042/). Among them, there are a total of 22 traditional Chinese medicine injections for the treatment of tumors. These injections can inhibit tumor and improve immunity, and have unique advantages in enhancing the sensitivity of radiotherapy and chemotherapy, relieving patients’ pain, improving patients’ quality of life, prolonging survival time, and so on.

Cinobufacini injection is a traditional Chinese medicine extract (Drug Approval Number: Z34020273, National Medical Products Administration), which has a long-term adjuvant effect on chemotherapy in patients with NSCLC.^[17–20]^ Recent studies have shown that cinobufacini can induce tumor cell apoptosis and downregulate the tumor-promoting inflammatory signal pathway in tumor microenvironment.^[21–23]^ In addition, cinobufacini injection can inhibit many kinds of human tumors such as liver cancer, gastric cancer, and pancreatic cancer.^[24–26]^ And cinobufacini injection is often used in combination with vinorelbine cisplatin chemotherapy in the treatment of stage III/IV NSCLC.^[27–29]^ However, the efficacy and safety of this combination therapy are still unclear. Since there is no systematic review of cinobufacini injection combined with vinorelbine cisplatin in the treatment of stage III/IV NSCLC, this systematic review and meta-analysis will conduct a high-quality comprehensive evaluation of its efficacy according to the preferred reporting item for systematic review and meta-analysis (PRISMA) statement.

## 2 Methods

### 2.1 Study registration

INPLASY registration number is INPLASY202060091. This systematic review (SR) will be reported following the PRISMA statement guidelines. If we improve the procedures described in this agreement, we will update our records in INPLASY and disclose them in future publications related to this study (https://inplasy.com/).

### 2.2 Inclusion criteria

#### 2.2.1 Types of studies

Only the randomized controlled trial (RCTs) of cinobufacini injection combined with Vinorelbine cisplatin for NSCLC will be included, regardless of language or publication status.

#### 2.2.2 Type of participants

Adult human populations (over 18 years old) who were pathologically diagnosed as NSCLC with clinical stages III (unresectable) and IV. Patients have no restrictions on gender, race, or country.

#### 2.2.3 Type of interventions

The experimental group received vinorelbine plus cisplatin chemotherapy plus cinobufacini injection. The control group only received vinorelbine plus cisplatin chemotherapy.

#### 2.2.4 Types of outcome measurements

The main outcome measures were based on the World Health Organization criteria for judging the efficacy of solid tumors or the objective criteria for evaluating the efficacy of solid tumors established by RECIST.^[30,31]^ The secondary criteria were KPS score, pain efficacy criteria, side effects of chemotherapy such as myelosuppression and gastrointestinal symptoms.

### 2.3 Exclusion criteria

The exclusion criteria include:

(1) Studies such as reviews, animal researches, observational studies without control group;
(2) Participants who had nonpathological diagnosis, previously subjected to chemotherapy, radiotherapy or surgery, concurrent infection, or other malignancies or severe illnesses;
(3) Participants in the control group who were treated with other antitumor traditional Chinese medicine (TCM) drugs;
(4) Repeatedly published literature or reviews;
(5) The use of acupuncture and other TCM therapies;
(6) Lung cancer is a metastatic focus of other tumors;
(7) Psychopath.

### 2.4 Search methods for the identification of studies

#### 2.4.1 Search strategy

We will carry out a comprehensive search of the PubMed, Web of Science, Cochrane Library, EMBASE, the Chinese Science and Technology Periodical Database, China National Knowledge Infrastructure, Wanfang Databases. The search strategies in Chinese and English are shown in Appendix 1 (https://kdocs.cn/l/sXPTh0nnD). Other sources will also be searched, including reference lists of identified publications and minutes of meetings. We will manually search for gray literature, including unpublished conference articles. The last search date will be July 30, 2020. Reference list of all selected articles will independently screened to identify additional studies left out in the initial search.

#### 2.4.2 Study selection

**Figure 1:**
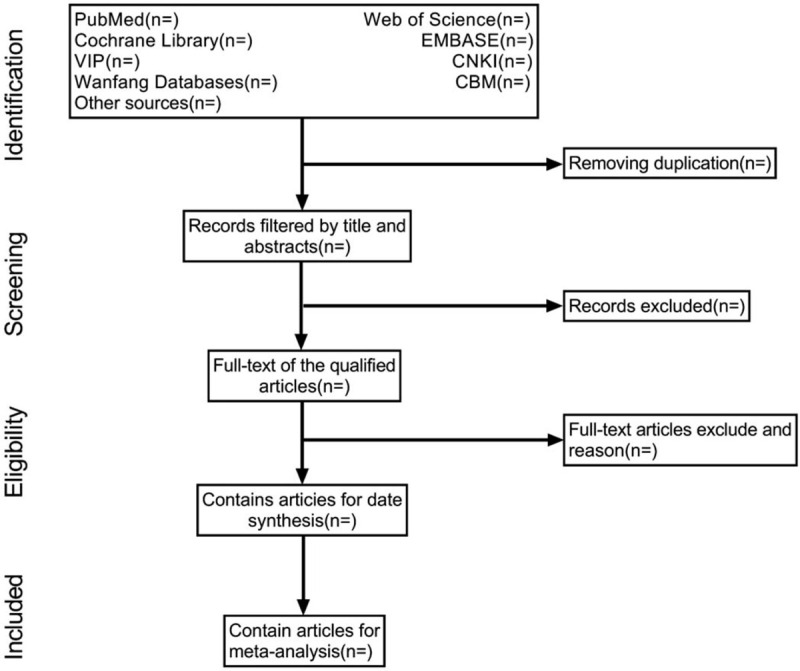
Flow diagram of studies identified.

All retrieved studies will be managed with Endnote X9, and repetitive studies will be filtered. Two reviewers (QL and LNZ) will independently screen the studies by titles and abstracts according to the predefined inclusion criteria. The 2 reviewers will then download the full text of all possible relevant studies and further review the full report independently. Two reviewers will cross check the included studies. Disagreements will be resolved by discussion or consensus with a 3rd reviewer (RLL). The procedures of study selection will be performed in accordance with the PRISMA flowchart (see Fig. 1).

#### 2.4.3 Data collection and management

Two reviewers (QL and QRY) will independently evaluate the included RCTs and extract the data. The following data will be extracted: author, year of publication, country in which the study was conducted, study cycle, original selection criteria, total number and age of participants in the study, course of disease, intervention measures, outcome indicators, and adverse events. Disagreements will be resolved by discussion or consensus with a 3rd reviewer (LNZ).

### 2.5 Assessment of risk of bias

The 2 reviewers (QRY and RLL) will use the Cochrane system evaluator's manual 5.1.0 bias risk assessment tool to evaluate the quality of the included literature, including whether the random method is correct, whether the allocation is hidden, the blind method, the completeness of the result data, whether to selectively report the research results, and whether there are other sources of bias.^[32]^ Each item is divided into low bias risk, high bias risk, and ambiguity. Any disagreement will be resolved through discussion among all authors.

### 2.6 Data analysis

We will use RevMan 5.3 software provided by Cochrane collaboration Network for meta-analysis. For dichotomous data, relative risk and 95% confidence interval (CI) were used to express, continuous variable data statistical method was expressed by mean difference and 95% CI, and the same outcome index was expressed by standardized mean difference and 95% CI if the measurement unit was different. The heterogeneity test among the included results was analyzed by *I*^2^ test: if there was no heterogeneity among studies (*P* > .1, *I*^2^ < 50%), fixed effect model was used for combined analysis; if there is heterogeneity among studies (*P* ≤ .1, *I*^2^ ≥ 50%), random effect model was used for combined analysis.

#### 2.6.1 Missing data management

If the included research data are insufficient or unclear, the original author will be contacted by email for more information. If there is no reply from the original author, we will only analyze the existing data. To investigate whether these missing data will affect the results of the meta-analysis, we will analyze the existing data and discuss the potential impact of the missing data.

#### 2.6.2 Subgroup analysis

In order to study heterogeneity, we will conduct a subgroup analysis based on age, sex, drug dose, and treatment time.

#### 2.6.3 Sensitivity analysis

In order to determine the robustness of the results of Meta analysis, we will conduct sensitivity analysis by excluding RCTs with high risk of deviation or RCTs with missing data.

#### 2.6.4 Reporting bias

If there are more than 10 studies in the meta-analysis, we will evaluate the symmetry of the funnel chart to test publication bias and carefully interpret the results.

#### 2.6.5 Confidence in cumulative evidence

The 2 reviewers will use the recommended evaluation, development, and evaluation system to independently evaluate the quality of evidence for each outcome.^[33]^ According to the 5 factors that may reduce the evidence quality in the scoring system (limitation, inaccuracy, inconsistency, indirect, publication bias), the evidence quality is divided into 4 grades: high, medium, low, and very low.

### 2.7 Ethics and dissemination

No ethical approval is required, as SR will be based on published research. According to the PRISMA guidelines, SR's results will be published in a peer-reviewed scientific journal.

## 3 Discussion

NSCLC is the most common lung cancer. Despite advances in early detection and standard treatment, NSCLC is frequently diagnosed at an advanced stage and therefore patients have a poor prognosis. Despite the introduction of innovative therapies, the 5-year survival of NSCLC is still <20%. We expect that the treatment of NSCLC in the future will be “comprehensive treatment.” The meta-analysis of high-quality trials will provide the most reliable evidence for the clinical treatment of NSCLC. The purpose of this systematic review and meta-analysis was to evaluate the efficacy and safety of cinobufacini injection combined with vinorelbine cisplatin in patients with stage III/IV NSCLC. We will comprehensively introduce the efficacy and adverse events of adjuvant therapy with cinobufacini injection. It is hoped that this result will help to establish a better method for the treatment of stage III/IV NSCLC and provide reliable evidence for its wide application.

### 3.1 Limitations

There may be a limitation with search, since only English and Chinese databases will be searched.

## Author contributions

All authors read and approved the final manuscript. Hua Xiao is the guarantor of the review.

**Data curation:** Ren-long Liang, Qian-Ru Yu, Li-Na Zhao.

**Methodology:** Hua Xiao, Xiao-Jia Yong, Xiao-dong Peng.

**Resources:** Wen-Wen Wang.

**Supervision:** De-Qing Tian.

**Writing – original draft:** Qian Li.

## Supplementary Material

Supplemental Digital Content
