# Supplementary material for: Efficacy and safety of cinobufacini injection combined with vinorelbine and cisplatin regimen chemotherapy for stage III/IV non-small cell lung cancer: A protocol for systematic review and meta-analysis of randomized controlled trials
Source: Medicine (Baltimore). 2020 Jul 31;99(31):e21539. doi: 10.1097/MD.0000000000021539 (PMC7402891; doi:10.1097/MD.0000000000021539)
Supplement: Supplemental Digital Content [file medi-99-e21539-s001.doc]

**Appendix 1**

**Search strategy applied in English databases and search strategy applied in Chinese databases.**

**Search strategy for Pubmed：**

#1 Non-small-cell-lung cancer[Mesh]

#2 Carcinoma, non-small-cell lung

#3 Non-small-cell lung carcinomas

#4 Non-small-cell lung carcinoma

#5 Lung carcinomas, non-small-cell

#6 Carcinoma, non-small cell lung

#7 Lung carcinomas, non-small-cell

#8 #1 OR #2 OR #3 OR #4 OR #5 OR #6 OR #7

#9 Cinobufacini injection

#10 huachansu injection

#11 #9 OR #10

#12 vinorelbine

#13 cisplatin

#14 #12 AND #13

#15 #8 AND #11 AND #14

**Search strategy for CNKI：**

#1 Feixiaoxibaofeiai (non-small-cell-lung cancer)

#2 Changchunruibin(vinorelbine)

#3 Shunbo(cisplatin)

#4 #2 AND #3

#5 Huachansuzhusheye (Cinobufacini injection)

#6 #1 AND #4 AND #5
